# Intranasal Lidocaine for Primary Headache Management in Emergency Department; a Clinical Trial

**Published:** 2017-09-16

**Authors:** Hassan Barzegari, Hassan Motamed, Behrad Ziapour, Majid Hajimohammadi, Mina Kadkhodazadeh

**Affiliations:** 1 Emergency Medicine Department, Ahvaz Jundishapur University of Medical Sciences, Ahvaz, Iran.

**Keywords:** Pain management, tension-type Headache, administration, intranasal, lidocaine, migraine disorders

## Abstract

**Introduction:**

Most of the headache cases only require pain management in emergency department (ED). The present study aimed to evaluate the efficacy of intranasal lidocaine in this regard.

**Method:**

In this clinical trial, adult patients with primary headache were randomly treated with 7.5 mg intravenous (IV) chlorpromazine and 1 ml intranasal lidocaine 2% (treatment) or normal saline 0.9% (placebo), and were compared 5, 15, and 30 minutes later regarding success rate using SPSS 21.

**Result:**

100 patients were assigned to either treatment or placebo group. Number needed to treat of intranasal lidocaine at 5, 15, and 30 minutes were 4 (95% CI: 2.2 – 6.6), 3 (95% CI: 1.7 – 3.5), and 4 (95% CI: 2.3 – 15.9), respectively. These measures for absolute risk reduction were 30 (95% CI: 15.2 – 44.8), 44 (95% CI: 28.7 – 59.3), and 26 percent (95% CI: 6.3 – 44.3), respectively. Pain relapse occurred in 16% of treatment and 11% of control group within 1 hour of treatment (p = 0.402).

**Conclusion:**

It seems that, intranasal lidocaine along with IV chlorpromazine could result in more successful and faster management of primary headaches in ED.

## Introduction

Headache is a common cause of emergency department (ED) visits. Most of these patients only require symptomatic treatment and referral to a neurologist or the patient’s family physician on an outpatient basis (1).

Migraine, tension, and cluster are three types of primary headaches with considerable clinical overlap, which suggests the same pathophysiology (1).

Common drugs used to treat these headaches are intravenous (IV) and oral opioids, ergot alkaloids, antiemetic, and non-steroidal anti-inflammatory drugs (NSAIDS) (2, 3). 

Intranasal route of drug administration may be more effective than other routes, with lower chance of emesis, and a quicker onset of action (3). Intranasal delivery of zolmitriptan, civamide, cocaine, and sumatriptan has been tried for treatment of migraine and cluster headaches (4-7).

Lawrence Robbins showed the safety of intranasal lidocaine as a an adjunctive medication in cluster headache control (8). Studies have reported that nasal lidocaine decreased cluster and migraine headaches within several seconds to 2 minutes (9, 10). However, Blanda and their colleagues didn’t find any evidence that supports the mentioned issue (11).

There has been limited experiences regarding primary headache management through intranasal medication in ED. Therefore, this study aimed to evaluate the efficacy of intranasal lidocaine in primary headache management in ED.

## Methods

***Study design and setting***

This randomized, double blind placebo controlled trial was done on patients presenting to emergency department of Golestan Hospital, Ahwaz, Iran, from July 2012, to December 2014 with primary headache (migraine, cluster or tension). The study design was approved by ethics committee of Ahvaz Jundishapur University of Medical Sciences under the number U-91159 and registered on Iranian Registry of Clinical Trials under this number: IRCT201212289148N2. Written informed consent was obtained from all patients. Researchers adhered to declaration of Helsinki protocol and confidentiality of patients’ information.

***Participants***

The subjects were patients between 15 – 55 years old who presented to the emergency department with complaints of primary headaches (migraine, cluster, or tension) according to the definition of International Headache Society and Ad Hoc Committee on Classification of Headache. Patients with signs of secondary headaches such as fever, meningismus, trauma, unstable vital signs, and altered mental status were excluded. Lactating and pregnant women as well as who had taken analgesic medications 2 hours before referring to ED were also excluded. Participants were randomly assigned to either treatment or control group using simple random sampling method.

***Intervention***

Patients in the treatment group received 1 mL intranasal lidocaine 2% (20 mg lidocaine) and 7.5 mg intravenous (IV) chlorpromazine and those in control group received 1 mL intranasal normal saline 0.9% and 7.5 mg IV chlorpromazine. The lidocaine and normal saline were sprayed with the same shape and color containers.

***Data gathering***

A checklist that consisted of baseline characteristics (sex, age), type of primary headache, pain severity, and possible complications was filled for all patients by a senior emergency medicine resident under supervision of an emergency medicine physician. Visual analog scale (VAS) was used to rate the pain severity at the baseline as well as 5, 15, and 30 minutes after drug administration. In addition, patients were observed for an additional 30 minutes to assess whether they responded to the treatment or whether their pain returned. 

Patients, in charge physicians, and data analyzer were blinded to drugs given to each group. 

***Outcome***

5, 15, and 30 minute success rates were considered as the main outcome of the study. 

***Statistical analysis***

Considering 95% confidence interval (CI), 80% power, and according to the Maizals et al. and Blanda et al. findings (11, 12), the minimum sample size for each study group was calculated to be 50 cases. Data analysis (intention to treat analysis) was performed by the statistical package for social sciences (SPSS) version 21. Findings were presented as mean ± standard deviation or frequency and percentage. Student t test, ANOVA and chi square or Fisher’s exact tests were used for comparisons. Success was defined as an at least 3 points decrease of pain severity (based on VAS) 5, 15, or 30 minutes after treatment. P < 0.05 was considered as significant. 

## Results

***Baseline characteristics***

100 patients were randomly assigned to either treatment (50 cases) or control (50 cases) group (54.0% female). The mean age of treatment and control groups were 32.96 ± 8.51 and 29.60 ± 8.64 years, respectively (p = 0.050). Table 1 shows the baseline characteristics of studied patients. 

***Pain control***

**Figure 1:**
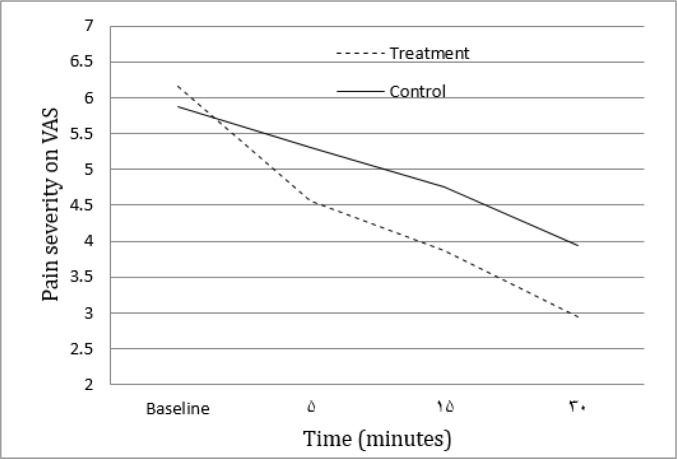
Pain severity at the baseline and 5, 15, and 30 minutes after treatment in lidocaine and placebo groups based on visual analogue scale (VAS).

The mean pain severity of patients at the time of presenting to emergency department and 5, 15, and 30 minutes after treatment are summarized in table 2 and figure 1. 

The success rates of two groups at 5, 15, and 30 minutes are compared in table 3. There was not any significant correlation between success rate and sex (p = 0.292), age (p = 0.380), and type of headache (p = 0.489).

Number needed to treat of intranasal lidocaine at 5, 15, and 30 minutes were 4 (95% CI: 2.2 – 6.6), 3 (95% CI: 1.7 – 3.5), and 4 (95% CI: 2.3 – 15.9), respectively. These measures for absolute risk reduction were 30 (95% CI: 15.2 – 44.8), 44 (95% CI: 28.7 – 59.3), and 26 percent (95% CI: 6.3 – 44.3), respectively.

The side effects related to treatment were not apparent in either the treatment or the control group within 30 minutes after treatment. Pain relapse occurred in 16% of the treatment and 11% of the control group during 1 hour follow up (p = 0.402).

## Discussion

Considering 30, 44, and 26 percent absolute risk reduction at 5, 15, and 30 minutes after treatment, intranasal lidocaine along with intravenous chlorpromazine could result in more successful pain management of primary headaches in ED. 

**Table 1:** Baseline characteristics of the two studied groups

**Variable**	**Group n (%)**		**P value**
	**Intranasal lidocaine**	**Placebo**	
**Sex**			
Male	22 (44.0)	24 (48.0)	0.688
Female	28(56.0)	26 (52.0)	
**Age (year)**			
20 – 29.9	20 (40.0)	31 (62.0)	0.038
30 – 39.9	10 (20.0)	10 (20.0)	
≥ 40	20 (40.0)	9 (18.0)	
**Type of headache**			
Migraine	16 (32.0)	22 (44.0)	0.028
Tension	11 (22.0)	18 (36.0)	
Cluster	23 (46.0)	10 920.0)	

**Table 2:** Pain severity of two studied groups based on visual analogue scale

**Time (minute)**	**Groups**		**P value**
	**Intranasal lidocaine**	**Placebo**	
Baseline	6.15 ± 1.19	5.87 ± 1.01	0.225
5	4.56 ± 1.54	5.30 ± 1.29	0.011
15	3.86 ± 1.57	4.76 ± 1.09	0.001
30	2.94 ± 1.63	3.94 ± 1.52	0.002

Data are presented as mean ± standard deviation.

**Table 3:** Success rate of the two studied groups 5, 15, and 30 minutes after treatment

**Time (minute)**	**Group n (%)**		**P value**
	**Intranasal lidocaine**	**Placebo**	
5	18 (36)	3 (6)	< 0.0001
15	25 (50)	3 (6)	< 0.0001
30	30 (60)	17 (34)	0.009

This study showed that patients who received intranasal lidocaine along with IV chlorpromazine significantly experienced higher success rate in pain control at 5, 15, and 30 minutes after treatment. 

Although the two studied groups had different baseline characteristics, the analysis of data didn’t show any relationship between age, sex, and type of primary headache with treatment success rate.

Maizels and Geiger evaluated the efficacy of intranasal lidocaine in a double-blind controlled trial with open-label follow-up. They found that headache was relieved within 15 min in 35.8% of patients in the treatment group, with a 20.6% relapse rate (12).

Mohammadkarimi and colleagues using intranasal lidocaine in 90 patients with primary and secondary headaches found significant pain relief after 1 minute, and showed that the level of patients’ pain did not significantly change over the course of the study. They did not assess the relapse rates or side effects related to treatment (9).

In contrast, Blanda and colleagues found that the intranasal lidocaine did not relieve pain after 5 or 30 minutes (11).

Although the experience of drug delivery through nasal mucosa goes back to many years ago, based on a review in 2013 the present method for delivering the drug to the posterior-superior part of the nose is not that effective and this could affect the success rate of this method to a great extent (13).

It seems that using a new method that can be applied by the patients themselves without the need for visiting the hospital should be considered more seriously. This is of great importance, especially for cases such as primary headaches that are usually recurrent and distractive. Of course, there is still a long way to go in making the use of these drugs public and revising traditional methods.

***Limitation***

In the current study, we administered intranasal 2% lidocaine for different types of headache and not just for migraine. We followed patients in both the treatment and control groups for only a short time. We did not perform follow-up assessments. In addition, because our treatment group received lidocaine and chlorpromazine together, our study is limited in its assessment of pure lidocaine’s ability in pain management.

## Conclusion

Considering 30, 44, and 26 percent absolute risk reduction at 5, 15, and 30 minutes after treatment, intranasal lidocaine along with IV chlorpromazine could result in more successful and faster management of primary headaches in ED. 
